# Supplementary material for: Analysis of CD74 Occurrence in Oncogenic Fusion Proteins
Source: Int J Mol Sci. 2023 Nov 5;24(21):15981. doi: 10.3390/ijms242115981 (PMC10650716; doi:10.3390/ijms242115981)
Supplement: Supplementary file 1 [file ijms-24-15981-s001.zip › ijms-2643806-supplementary.pdf]

SUPPORTING INFORMATION

Analysis of CD74 Occurrence in Oncogenic Fusion Proteins

Jasmine Vargas and Georgios Pantouris *

Department of Chemistry, University of the Pacific, Stockton, CA 95211, USA
* Correspondence: gpantouris@pacific.edu

Table of Contents

Tables

Table S1: Retrospective studies on tumor samples containing the CD74-ROS1 fusion.

Table S2: Retrospective studies on tumor samples containing the CD74-NTRK1 fusion.

Table S3: Retrospective studies on tumor samples containing the CD74-NRG1 fusion.

Table S4: Retrospective study on a tumor sample containing the CD74-PDGFRB fusion.

Table S5: Retrospective study on a tumor sample containing the CD74-NRG2α fusion.

Figures

Figure S1: Amino acid sequences of CD74 isoforms.

Figure S2: Identified CD74 oncogenic fusions in the human body.

Figure S3: Previously reported amino acid sequences of CD74 fusion proteins.

Table S1: Retrospective studies on tumor samples containing the CD74-ROS1 fusion.

Diagnosis	Variant	Specimen	Detection Method	Age	Gender	Smoker/ Pack Year (PY)	Stage	Reference
NSCLC	C6-R34	FFPE tumor tissue	FISH, RT-PCR	50	F	0	IB	[1]
NSCLC	C6-R34	FFPE tumor tissue	FISH, RT-PCR	53	M	0	IIA	[2]
NSCLC	C6-R34	FFPE tumor tissue	FISH, RT-PCR	51	F	0	IIIB	[2]
NSCLC	C6-R34	FFPE tumor tissue	FISH, RT-PCR	55	F	23	IV	[2]
NSCLC	C6-R34	FFPE tumor tissue	FISH, RT-PCR	44	F	-	IV	[2]
NSCLC	C6-R34	FFPE tumor tissue	FISH, RT-PCR	57	F	0	IV	[2]
NSCLC	C6-R34	Tumor TMA Snap-frozen tumor sample	FISH, RT-PCR	57	M	1480	IA	[3]
NSCLC	C6-R34	Tumor TMA Snap-frozen tumor sample	FISH, RT-PCR	60	M	930	IIIB	[3]
NSCLC	C6-R34 C6-R32	Tumor TMA Snap-frozen tumor sample	FISH, RT-PCR	79	F	0	IA	[3]
NSCLC	C6-R34	FFPE tumor tissue	Multiplex RTPCR	65	F	0	IIB	[4]
NSCLC	C6-R32	FFPE tumor tissue	Multiplex RTPCR	44	F	0	IV	[4]
NSCLC	C6-R34	FFPE tumor tissue	Multiplex RTPCR	49	F	0	IA	[4]
NSCLC	-	FFPE tumor tissue	NGS	77	F	0	IB	[5]
NSCLC	-	Fresh frozen tumor tissue	WGS RNA seq	58	M	4.3	-	[6]
NSCLC	-	Fresh frozen tumor tissue	WGS RNA seq	46	F	0	-	[6]
NSCLC	-	Fresh frozen tumor tissue	WGS RNA seq	48	M	25	-	[6]
NSCLC	-	Fresh frozen tumor tissue	WGS RNA seq	57	M	14	-	[6]
NSCLC	-	Fresh frozen tumor tissue	WGS RNA seq	74	M	13	-	[6]

NSCLC	C6-R34	Frozen tumor tissue	RT-PCR	66	F	0	IIIA	[7]
NSCLC	C6-R34	Frozen tumor tissue	RT-PCR	48	F	0	IIIA	[7]
NSCLC	C6-R34	Frozen tumor tissue	RT-PCR	50	F	0	I	[7]
NSCLC	C6-R34	Frozen tumor tissue	RT-PCR	51	F	0	I	[7]
NSCLC	C6-R34	Frozen tumor tissue	RT-PCR	42	M	Yes	I	[7]
NSCLC	C6-R34	Tissue biopsy, blood, or pleural effusion	NGS	58	M	Former	IV	[8]
NSCLC	C6-R34	Tissue biopsy, blood, or pleural effusion	NGS	52	F	0	IV	[8]
NSCLC	C6-R34	Tissue biopsy, blood, or pleural effusion	NGS	47	F	0	III	[8]
NSCLC	C6-R34	Tissue biopsy, blood, or pleural effusion	NGS	37	F	0	IV	[8]
NSCLC	C6-R34	Tissue biopsy, blood, or pleural effusion	NGS	62	M	Former	IV	[8]
NSCLC	C6-R34	Tissue biopsy, blood, or pleural effusion	NGS	42	M	Former	IV	[8]
NSCLC	C6-R34	Tissue biopsy, blood, or pleural effusion	NGS	71	F	Former	IV	[8]
NSCLC	C6-R34	Tissue biopsy, blood, or pleural effusion	NGS	56	F	Former	IV	[8]
NSCLC	-	Tissue biopsy, blood, or pleural effusion	NGS	49	M	-	IV	[8]
NSCLC	-	Tissue biopsy, blood, or pleural effusion	NGS	48	F	-	IV	[8]

NSCLC	-	Tissue biopsy, blood, or pleural effusion	NGS	49	F	-	IV	[8]
NSCLC	-	Tissue biopsy, blood, or pleural effusion	NGS	49	F	-	IV	[8]
NSCLC	-	Tissue biopsy, blood, or pleural effusion	NGS	30	F	-	IV	[8]
NSCLC	-	Tissue biopsy, blood, or pleural effusion	NGS	50	F	-	IV	[8]
NSCLC	-	Tissue biopsy, blood, or pleural effusion	NGS	29	F	-	IV	[8]
NSCLC	C6-R34	FFPE tumor tissue	Multiplex RT-PCR IHC	44	M	0	II	[9]
NSCLC	C6-R34	FFPE tumor tissue	Multiplex RT-PCR IHC	45	F	0	IIIA	[9]
NSCLC	C6-R34	FFPE tumor tissue	Multiplex RT-PCR IHC	37	F	0	II	[9]
NSCLC	C6-R34	FFPE tumor tissue	Multiplex RT-PCR IHC	59	F	0	IV	[9]
NSCLC	C6-R34	FFPE tumor tissue	Multiplex RT-PCR IHC	46	M	10	IV	[9]
NSCLC	C6-R34	FFPE tumor tissue	Multiplex RT-PCR IHC	36	M	5	IV	[9]
NSCLC	C6-R34	FFPE tumor tissue	Multiplex RT-PCR IHC	45	M	17	IV	[9]
NSCLC	C6-R34	FFPE tumor tissue	Multiplex RT-PCR IHC	32	M	15	IV	[9]

NSCLC	C6-R34<br>C6-R32	FFPE tumor tissue	Multiplex RT-PCR<br>IHC	59	M	5	IV	[9]
NSCLC	C6-R34	FFPE tumor tissue	FISH RT-PCR	55	M	0	-	[10]
NSCLC	C6-R34	FFPE tumor tissue	FISH RT-PCR	67	F	0	-	[10]
NSCLC	C6-R34	TMA<br>FFPE tumor tissue	FISH<br>RT-PCR<br>Inverse PCR	71	M	Current smoker	I	[11]
NSCLC	C6-R34	TMA<br>FFPE tumor tissue	FISH<br>RT-PCR<br>Inverse PCR	41	F	0	III	[11]
NSCLC	C6-R34	FFPE tumor tissue	NGS	-	-	-	-	[12]
NSCLC	C6-R34	FFPE tumor tissue	NGS	-	-	-	-	[12]
NSCLC	C6-R34	FFPE tumor tissue	NGS	-	-	-	-	[12]
NSCLC	C6-R34	Malignant Pleural Effusion	NGS	-	-	-	-	[12]
NSCLC	C7-R34	FFPE tumor tissue	NGS	-	-	-	-	[12]
NSCLC	C6-R34	FFPE tumor tissue	NGS	-	-	-	-	[12]
NSCLC	C7-R34	FFPE tumor tissue	NGS	-	-	-	-	[12]
NSCLC	C7-R34	FFPE tumor tissue	NGS	-	-	-	-	[12]
NSCLC	C7-R34	FFPE tumor tissue	NGS	-	-	-	-	[12]
NSCLC	C6-R33	FFPE tumor tissue	NGS	-	-	-	-	[12]
NSCLC	C6-R32	Pleural effusion	nCounter (Nanostring)	-	-	-	-	[13]
NSCLC	C6-R32	Plasma	nCounter (Nanostring)<br>dPCR	-	-	-	-	[13]
NSCLC	C6-R32	Plasma	nCounter (Nanostring)	-	-	-	-	[13]
NSCLC	C6-R34	Frozen tumor tissue	RT-PCR<br>FISH	55	F	0	IA	[14]
NSCLC	C6-R34	Frozen tumor tissue	RT-PCR<br>FISH	40	F	0	IA	[14]
NSCLC	C6-R34	Frozen tumor tissue	RT-PCR<br>FISH	47	M	0	IIIB	[14]

NSCLC	C6-R34	Frozen tumor tissue	RT-PCR FISH	64	F	0	IIIA	[14]
NSCLC	C6-R34	Frozen tumor tissue	RT-PCR FISH	59	F	0	IA	[14]
NSCLC	C6-R34	Frozen tumor tissue	RT-PCR FISH	68	F	0	IA	[14]
NSCLC	C6-R34	Frozen tumor tissue	RT-PCR FISH	61	F	0	IIIA	[14]
NSCLC	C6-R34	Frozen tumor tissue	RT-PCR FISH	66	F	0	IA	[14]
NSCLC	C6-R34	Frozen tumor tissue	RT-PCR FISH	59	M	11	IIIA	[14]
NSCLC	C6-R34	Frozen tumor tissue	RT-PCR FISH	37	F	0	IIA	[14]

NSCLC: non-small cell lung cancer, NGS: next-generation sequencing, FISH: fluorescence in situ hybridization, RT-PCR: reverse transcription-polymerase chain reaction, dPCR: digital polymerase chase reaction, IHC: immunohistochemistry, WGS: whole-genome sequencing, TMA: tissue microarray, Seq: sequencing, FFPE: formalin-fixed paraffin-embedded, - indicates data not available.

Table S2: Retrospective studies on tumor samples containing the CD74-NTRK1 fusion.

Diagnosis	Variant	Specimen	Detection Method	Age	Gender	Stage	Reference
NSCLC	C8-N12	FFPE or frozen tumor sample	NGS RT-PCR FISH	31-83	-	-	[15]
NSCLC	C7-N8	FFPE	NGS	41	F	IIIB	[16]
NSCLC	C6-N12	Tissue, whole blood, or pleural effusion	NGS	34-79	-	-	[17]
NSCLC	-	Tumor tissue	IHC RNA seq	62	F	-	[18]

NSCLC: non-small cell lung cancer, FFPE: formalin-fixed paraffin-embedded, NGS: next generation sequencing, RT-PCR: reverse transcription-polymerase chain reaction, FISH: fluorescence in situ hybridization, IHC: immunohistochemistry, Seq: sequencing, - indicates data not available.

Table S3: Retrospective studies on tumor samples containing the CD74-NRG1 fusion.

Diagnosis	Variant	Specimen	Detection Method	Age	Gender	Smoker/ Pack Year (PY)	Stage	Reference
IMA	C6-N6	Fresh frozen tumor tissue FFPE tumor tissue	Chromosomal gene copy number analysis Transcriptome sequencing FISH NGS RT-PCR	64	F	0	IB	[19]
IMA	C6-N6	Snap-frozen tissue sample	Whole transcriptome sequencing (RNA seq) RT-PCR Sanger sequencing	68	F	0	2B	[20]
IMA	C6-N6	Snap-frozen tissue sample	Whole transcriptome sequencing (RNA seq) RT-PCR Sanger sequencing	53	F	0	IA	[20]
IMA	C8-N6	Snap-frozen tissue sample	Whole transcriptome sequencing (RNA seq) RT-PCR Sanger sequencing	55	M	47	IA	[20]
IMA	C8-N6	Snap-frozen tissue sample	Whole transcriptome sequencing (RNA seq) RT-PCR Sanger sequencing	78	F	0	IA	[20]

IMA	C8-N6	Snap-frozen tissue sample	Whole transcriptome sequencing (RNA seq) RT-PCR	47	F	0	IB	[20]
			Sanger sequencing					
NSCLC	C6-N4	FFPE tumor tissue	NGS	83	F	-	unknown	[21]
NSCLC	C6-N4	FFPE tumor tissue	NGS	80	F	-	unknown	[21]
IMA	-	FFPE tissue	mRNA NGS RT-PCR Sanger Sequencing	81	F	-	-	[22]
IMA	-	FFPE tissue	mRNA NGS RT-PCR Sanger Sequencing	60	F	-	-	[22]
IMA	-	Fresh frozen tumor sample FFPE tissue	RNA seq	70	F	100 smoking index	IA (pT1aN0M0)	[23]
IMA	-	Fresh frozen tumor sample FFPE tissue	RNA seq	63	F	0	IA (pT1aN0M0)	[23]

NSCLC: non-small cell lung cancer, IMA: invasive mucinous adenocarcinoma, FFPE: formalin-fixed paraffin-embedded, NGS: next generation sequencing, RT-PCR: reverse transcription-polymerase chain reaction, FISH: fluorescence in situ hybridization, IHC: immunohistochemistry, Seq: sequencing, - indicates data not available.

Table S4: Retrospective study on a tumor sample containing the CD74-PDGFRB fusion.

Diagnosis	Variant	Specimen	Detection Method	Age	Gender	OS	Reference
B-ALL	C6-P11	Bone marrow	RNA seq	2.4	M	CR after 2 courses of induction therapy	[24]

B-ALL: B-cell acute lymphoblastic leukemia, RNA seq: RNA sequencing, CR: complete response

Table S5: Retrospective study on a tumor sample containing the CD74-NRG2α fusion.

Diagnosis	Variant	Specimen	Detection Method	Age	Gender	Smoker/ Pack Year (PY)	Stage	Treatment	OS	Reference
Acinar Adenocarc inoma	C6-N2	Fresh frozen tumor sample FFPE tissue	RNA seq Sanger Seq	70	F	0	IIIA (pT2aN2M0)	erlotinib	Dead of disease (32 weeks)	[23]

FFPE: formalin-fixed paraffin-embedded, seq: sequencing

p33: 216 amino acids (Claesson et al. 1983)

MDDQRDLISNNEQLPMLGRRPGAPESKCSRGALYTGFSILVTLLLAGQATTAYFLYQQQGRLDKL
TVTSQNLQLENLRMKLPKPPKPVSKMRMATPLLMQALPMGALPQGPMQNATKYGNMTEDHVM
HLLQNADPLKVYPPLKGSFPENLRHLKNTMETIDWKVFESWMHHWLLFEMSRHSLEQKPTDAPPK
ESLELEDPSSGLGVTKQDLGPVPM

p35: 232 amino acids (Strubin et al. 1984)

MHRRRSRSCREDQKPVMDDQRDLISNNEQLPMLGRRPGAPESKCSRGALYTGFSILVTLLLAGQA
TTAYFLYQQQGRLDKLTVTSQNLQLENLRMKLPKPPKPVSKMRMATPLLMQALPMGALPQGPM
QNATKYGNMTEDHVMHLLQNADPLKVYPPLKGSFPENLRHLKNTMETIDWKVFESWMHHWLLF
EMSRHSLEQKPTDAPPKESLELEDPSSGLGVTKQDLGPVPM

p41: 280 amino acids (Strubin et al. 1986)

MDDQRDLISNNEQLPMLGRRPGAPESKCSRGALYTGFSILVTLLLAGQATTAYFLYQQQGRLDKL
TVTSQNLQLENLRMKLPKPPKPVSKMRMATPLLMQALPMGALPQGPMQNATKYGNMTEDHVM
HLLQNADPLKVYPPLKGSFPENLRHLKNTMETIDWKVFESWMHHWLLFEMSRHSLEQKPTDAPPK
VLTKCQEEVSHIPAVHPGSFRPKCDENGNYLPLQCYGSIGYCWCVFPNGTEVPNTRSRGHHNCSES
LELEDPSSGLGVTKQDLGPVPM

p43: 296 amino acids (O'Sullivan et al. 1986)

MHRRRSRSCREDQKPVMDDQRDLISNNEQLPMLGRRPGAPESKCSRGALYTGFSILVTLLLAGQA
TTAYFLYQQQGRLDKLTVTSQNLQLENLRMKLPKPPKPVSKMRMATPLLMQALPMGALPQGPM
QNATKYGNMTEDHVMHLLQNADPLKVYPPLKGSFPENLRHLKNTMETIDWKVFESWMHHWLLF
EMSRHSLEQKPTDAPPKVLTKCQEEVSHIPAVHPGSFRPKCDENGNYLPLQCYGSIGYCWCVFPNG
TEVPNTRSRGHHNCSESLELEDPSSGLGVTKQDLGPVPM

Figure S1: Amino acid sequences of CD74 isoforms. Amino acid count for p33 [25], p35 [26], p41 [27], and p43 [28] are included.

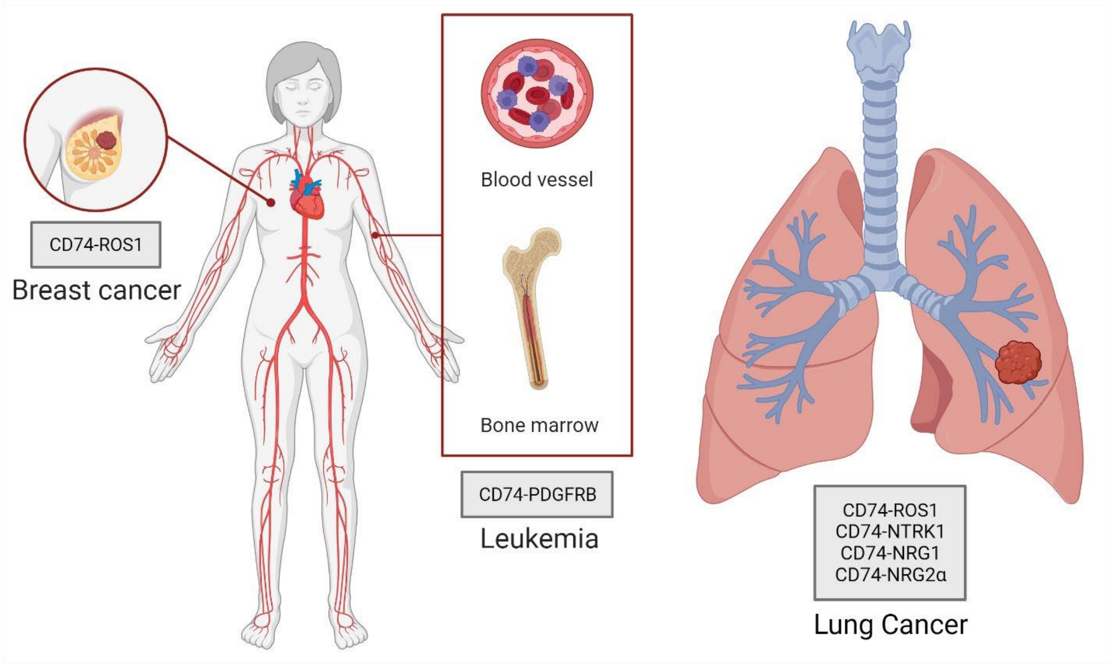

Figure S2. Identified CD74 oncogenic fusions in the human body

CD74-ROS1 C6-R34

703 aa (Rikova et al. 2007) (Takeuchi et al. 2012)

MHRRRSRSCREDQKPVMDDQRDLISNNEQLPMLGRRPGAPESKCSRGALYTGFSILVTLLLAGQATTAYFLYQ
QQGRLDKLTVTSQNLQLENLRMKLPKPPKPVSKMRMATPLLMQALPMGALPQGPMQNATKYGNMTEDHV
MHLLQNADPLKVYPPLKGSFPENLRHLKNTMETIDWKVFESWMHHWLLFEMSRHSLEQKPTDAPPKDDFWIP
ETSFILTIIVGIFLVVTIPLTFVWHRRLKNQKSAKEGVTVLINEDKELAELRGLAAGVGLANACYAIHTLPTQEEI
ENLPAFPREKLTLRLLLGSGAFGEVYEGTAVDILGVGSGEIKVAVKTLKKGSTDQEKIEFLKEAHLMSKFNHPNI
LKQLGVCLLNEPQYIILELMEGGDLLTYLRKARMATFYGPLLTLVDLVDLCVDISKGCVYLERMHFIHRDLAAR
NCLVSVKDYTSPRIVKIGDFGLARDIYKNDYYRKRGEGLLPVRWMAPESLMDGIFTTQSDVWSFGILIWEILTL
GHQPYPAHSNLDVLNYVQTGGRLEPPRNCPDDLWNLMTQCWAQEPDQRPTFHRIQDQLQLFRNFFLNSIYKS
RDEANNSGVINESFEGEDGDVICLNSDDIMPVALMETKNREGLNYMVLATECGQGEEKSEGPLGSQESESCG
LRKEEKEPHADKDFCQEKQVAYCPSGKPEGLNYACLTHSGYGDGSD

CD74-ROS1 C6-R32

806 aa (Takeuchi et al. 2012)

MHRRRSRSCREDQKPVMDDQRDLISNNEQLPMLGRRPGAPESKCSRGALYTGFSILVTLLLAGQATTAYFLYQ
QQGRLDKLTVTSQNLQLENLRMKLPKPPKPVSKMRMATPLLMQALPMGALPQGPMQNATKYGNMTEDHV
MHLLQNADPLKVYPPLKGSFPENLRHLKNTMETIDWKVFESWMHHWLLFEMSRHSLEQKPTDAPPKAGVPN
KPGIPKLLEGSKNSIQWEKAEDNGCRITYYILEIRKSTSNNLQNQNLRWKMTFNGSCSSVCTWKSKNLKGIFQF
RVVAANNLGFGEYSGISENIILVGDDFWIPETSFILTIIVGIFLVVTIPLTFVWHRRLKNQKSAKEGVTVLINEDK
ELAELRGLAAGVGLANACYAIHTLPTQEEIENLPAFPREKLTLRLLLGSGAFGEVYEGTAVDILGVGSGEIKVAV
KTLKKGSTDQEKIEFLKEAHLMSKFNHPNILKQLGVCLLNEPQYIILELMEGGDLLTYLRKARMATFYGPLLTL
VDLVDLCVDISKGCVYLERMHFIHRDLAARNCLVSVKDYTSPRIVKIGDFGLARDIYKNDYYRKRGEGLLPVR
WMAPESLMDGIFTTQSDVWSFGILIWEILTLGHQPYPAHSNLDVLNYVQTGGRLEPPRNCPDDLWNLMTQC
WAQEPDQRPTFHRIQDQLQLFRNFFLNSIYKSRDEANNSGVINESFEGEDGDVICLNSDDIMPVALMETKNRE
GLNYMVLATECGQGEEKSEGPLGSQESESCGLRKEEKEPHADKDFCQEKQVAYCPSGKPEGLNYACLTHSGY
GDGSD

CD74-NTRK1 C8-N12

627 aa (Vaishnavi et al. 2013)

MHRRRSRSCREDQKPVMDDQRDLISNNEQLPMLGRRPGAPESKCSRGALYTGFSILVTLLLAGQATTAYFLYQ
QQGRLDKLTVTSQNLQLENLRMKLPKPPKPVSKMRMATPLLMQALPMGALPQGPMQNATKYGNMTEDHV
MHLLQNADPLKVYPPLKGSFPENLRHLKNTMETIDWKVFESWMHHWLLFEMSRHSLEQKPTDAPPKESLELE
DPSSGLGVTKQDLGPDTNSTSGDPVEKKDETPFGVSVAVGLAVFACLFLSTLLLVLNKCGRRNKFGINRPAVL
APEDGLAMSLHFMTLGGSSLSPTEGKGSGLQGHIIENPQYFSDACVHHIKRRDIVLKWELGEGAFGKVFLAEC
HNLLPEQDKMLVAVKALKEASESARQDFQREAELLTMLQHQHIVRFFGVCTEGRPLLMVFEYMRHGDLNRFL
RSHGPDAKLLAGGEDVAPGPLGLGQLLAVASQVAAGMVYLAGLHFVHRDLATRNCLVGQGLVVKIGDFGM
SRDIYSTDYYRVGGRTMLPIRWMPPESILYRKFTTESDVWSFGVVLWEIFTYGKQPWYQLSNTEAIDCITQGRE
LERPRACPPEVYAIMRGCWQREPQQRHSIKDVHARLQALAQAPPVYLDVLG

Figure S3: Previously reported amino acid sequences of CD74 fusion proteins. CD74-ROS1 and CD74-NTRK1 cDNA sequences translated with Expasy Translate. Amino acid counts for CD74-ROS1 fusion variants C6-R34 [1] [3], C6-R32 [3], and CD74-NTRK1 fusion variant C8-N12 [15] are included.

References

1. Rikova, K.; Guo, A.; Zeng, Q.; Possemato, A.; Yu, J.; Haack, H.; Nardone, J.; Lee, K.; Reeves, C.; Li, Y.; Hu, Y.; Tan, Z.; Stokes, M.; Sullivan, L.; Mitchell, J.; Wetzel, R.; Macneill, J.; Ren, J. M.; Yuan, J.; Bakalarski, C. E.; Villen, J.; Kornhauser, J. M.; Smith, B.; Li, D.; Zhou, X.; Gygi, S. P.; Gu, T. L.; Polakiewicz, R. D.; Rush, J.; Comb, M. J., Global survey of phosphotyrosine signaling identifies oncogenic kinases in lung cancer. *Cell* **2007,** 131, (6), 1190-203.
2. Bergethon, K.; Shaw, A. T.; Ou, S.-H. I.; Katayama, R.; Lovly, C. M.; McDonald, N. T.; Massion, P. P.; Siwak-Tapp, C.; Gonzalez, A.; Fang, R.; Mark, E. J.; Batten, J. M.; Chen, H.; Wilner, K. D.; Kwak, E. L.; Clark, J. W.; Carbone, D. P.; Ji, H.; Engelman, J. A.; MinoKenudson, M.; Pao, W.; Iafrate, A. J., ROS1 Rearrangements Define a Unique Molecular Class of Lung Cancers. *Journal of Clinical Oncology* **2012,** 30, (8), 863-870.
3. Takeuchi, K.; Soda, M.; Togashi, Y.; Suzuki, R.; Sakata, S.; Hatano, S.; Asaka, R.; Hamanaka, W.; Ninomiya, H.; Uehara, H.; Lim Choi, Y.; Satoh, Y.; Okumura, S.; Nakagawa, K.; Mano, H.; Ishikawa, Y., RET, ROS1 and ALK fusions in lung cancer. *Nat Med* **2012,** 18, (3), 378-81.
4. Cai, W.; Li, X.; Su, C.; Fan, L.; Zheng, L.; Fei, K.; Zhou, C.; Manegold, C.; Schmid-Bindert, G., ROS1 fusions in Chinese patients with non-small-cell lung cancer. *Ann Oncol* **2013,** 24, (7), 1822-1827.
5. Drilon, A.; Wang, L.; Arcila, M. E.; Balasubramanian, S.; Greenbowe, J. R.; Ross, J. S.; Stephens, P.; Lipson, D.; Miller, V. A.; Kris, M. G.; Ladanyi, M.; Rizvi, N. A., Broad, Hybrid Capture-Based Next-Generation Sequencing Identifies Actionable Genomic Alterations in Lung Adenocarcinomas Otherwise Negative for Such Alterations by Other Genomic Testing Approaches. *Clin Cancer Res* **2015,** 21, (16), 3631-9.
6. Lee, J. J.; Park, S.; Park, H.; Kim, S.; Lee, J.; Lee, J.; Youk, J.; Yi, K.; An, Y.; Park, I. K.; Kang, C. H.; Chung, D. H.; Kim, T. M.; Jeon, Y. K.; Hong, D.; Park, P. J.; Ju, Y. S.; Kim, Y. T., Tracing Oncogene Rearrangements in the Mutational History of Lung Adenocarcinoma. *Cell* **2019,** 177, (7), 1842-1857.e21.
7. Chen, Y.; Huang, Y.; Ning, H.; Chen, X.; Tan, X.; Ding, X., Clinic-pathologic features and gene fusion pattern of ALK and ROS1 in non-small cell lung cancer show association with household coal combustion. *Transl Cancer Res* **2019,** 8, (5), 2164-2174.
8. Zhang, Y.; Zhang, X.; Zhang, R.; Xu, Q.; Yang, H.; Lizaso, A.; Xu, C.; Liu, J.; Wang, W.; Ou, S. I.; Zhang, J.; Song, Z.; Yang, N., Clinical and molecular factors that impact the efficacy of first-line crizotinib in ROS1-rearranged non-small-cell lung cancer: a large multicenter retrospective study. *BMC Med* **2021,** 19, (1), 206.
9. Chen, Y. F.; Hsieh, M. S.; Wu, S. G.; Chang, Y. L.; Shih, J. Y.; Liu, Y. N.; Tsai, M. F.; Tsai, T. H.; Yu, C. J.; Yang, J. C.; Yang, P. C., Clinical and the prognostic characteristics of lung adenocarcinoma patients with ROS1 fusion in comparison with other driver mutations in East Asian populations. *J Thorac Oncol* **2014,** 9, (8), 1171-9.
10. Kim, H. R.; Lim, S. M.; Kim, H. J.; Hwang, S. K.; Park, J. K.; Shin, E.; Bae, M. K.; Ou, S. H.; Wang, J.; Jewell, S. S.; Kang, D. R.; Soo, R. A.; Haack, H.; Kim, J. H.; Shim, H. S.; Cho, B. C., The frequency and impact of ROS1 rearrangement on clinical outcomes in never smokers with lung adenocarcinoma. *Ann Oncol* **2013,** 24, (9), 2364-70.
11. Davies, K. D.; Le, A. T.; Theodoro, M. F.; Skokan, M. C.; Aisner, D. L.; Berge, E. M.; Terracciano, L. M.; Cappuzzo, F.; Incarbone, M.; Roncalli, M.; Alloisio, M.; Santoro, A.; Camidge, D. R.; Varella-Garcia, M.; Doebele, R. C., Identifying and targeting ROS1 gene fusions in non-small cell lung cancer. *Clin Cancer Res* **2012,** 18, (17), 4570-9.
12. Zeng, L.; Li, Y.; Xiao, L.; Xiong, Y.; Liu, L.; Jiang, W.; Heng, J.; Qu, J.; Yang, N.; Zhang, Y., Crizotinib presented with promising efficacy but for concomitant mutation in nextgeneration sequencing-identified ROS1-rearranged non-small-cell lung cancer. *Onco Targets Ther* **2018,** 11, 6937-6945.
13. Giménez-Capitán, A.; Sánchez-Herrero, E.; Robado de Lope, L.; Aguilar-Hernández, A.; Sullivan, I.; Calvo, V.; Moya-Horno, I.; Viteri, S.; Cabrera, C.; Aguado, C.; Armiger, N.; Valarezo, J.; Mayo-de-Las-Casas, C.; Reguart, N.; Rosell, R.; Provencio, M.; Romero, A.; Molina-Vila, M. A., Detecting ALK, ROS1 and RET fusions and the METΔex14 splicing variant in liquid biopsies of non-small cell lung cancer patients using RNA-based techniques. *Mol Oncol* **2023**.

14. Yoshida, A.; Kohno, T.; Tsuta, K.; Wakai, S.; Arai, Y.; Shimada, Y.; Asamura, H.; Furuta, K.; Shibata, T.; Tsuda, H., ROS1-rearranged lung cancer: a clinicopathologic and molecular study of 15 surgical cases. *Am J Surg Pathol* **2013,** 37, (4), 554-62.
15. Vaishnavi, A.; Capelletti, M.; Le, A. T.; Kako, S.; Butaney, M.; Ercan, D.; Mahale, S.; Davies, K. D.; Aisner, D. L.; Pilling, A. B.; Berge, E. M.; Kim, J.; Sasaki, H.; Park, S.; Kryukov, G.; Garraway, L. A.; Hammerman, P. S.; Haas, J.; Andrews, S. W.; Lipson, D.; Stephens, P. J.; Miller, V. A.; Varella-Garcia, M.; Janne, P. A.; Doebele, R. C., Oncogenic and drug-sensitive NTRK1 rearrangements in lung cancer. *Nat Med* **2013,** 19, (11), 1469-1472.
16. Xia, H.; Xue, X.; Ding, H.; Ou, Q.; Wu, X.; Nagasaka, M.; Shao, Y. W.; Hu, X.; Ou, S. I., Evidence of NTRK1 Fusion as Resistance Mechanism to EGFR TKI in EGFR+ NSCLC: Results From a Large-Scale Survey of NTRK1 Fusions in Chinese Patients With Lung Cancer. *Clin Lung Cancer* **2020,** 21, (3), 247-254.
17. Li, H.; Yan, S.; Liu, Y.; Ma, L.; Liu, X.; Liu, Y.; Cheng, Y., Analysis of NTRK mutation and clinicopathologic factors in lung cancer patients in northeast China. *Int J Biol Markers* **2020,** 35, (3), 36-40.
18. Koopman, B.; Kuijpers, C.; Groen, H. J. M.; Timens, W.; Schuuring, E.; Willems, S. M.; van Kempen, L. C., Detection of NTRK Fusions and TRK Expression and Performance of panTRK Immunohistochemistry in Routine Diagnostics: Results from a Nationwide Community-Based Cohort. *Diagnostics (Basel)* **2022,** 12, (3).
19. Fernandez-Cuesta, L.; Plenker, D.; Osada, H.; Sun, R.; Menon, R.; Leenders, F.; OrtizCuaran, S.; Peifer, M.; Bos, M.; Dassler, J.; Malchers, F.; Schottle, J.; Vogel, W.; Dahmen, I.; Koker, M.; Ullrich, R. T.; Wright, G. M.; Russell, P. A.; Wainer, Z.; Solomon, B.; Brambilla, E.; Nagy-Mignotte, H.; Moro-Sibilot, D.; Brambilla, C. G.; Lantuejoul, S.; Altmuller, J.; Becker, C.; Nurnberg, P.; Heuckmann, J. M.; Stoelben, E.; Petersen, I.; Clement, J. H.; Sanger, J.; Muscarella, L. A.; la Torre, A.; Fazio, V. M.; Lahortiga, I.; Perera, T.; Ogata, S.; Parade, M.; Brehmer, D.; Vingron, M.; Heukamp, L. C.; Buettner, R.; Zander, T.; Wolf, J.; Perner, S.; Ansen, S.; Haas, S. A.; Yatabe, Y.; Thomas, R. K., CD74-NRG1 fusions in lung adenocarcinoma. *Cancer Discov* **2014,** 4, (4), 415-22.
20. Nakaoku, T.; Tsuta, K.; Ichikawa, H.; Shiraishi, K.; Sakamoto, H.; Enari, M.; Furuta, K.; Shimada, Y.; Ogiwara, H.; Watanabe, S.; Nokihara, H.; Yasuda, K.; Hiramoto, M.; Nammo, T.; Ishigame, T.; Schetter, A. J.; Okayama, H.; Harris, C. C.; Kim, Y. H.; Mishima, M.; Yokota, J.; Yoshida, T.; Kohno, T., Druggable oncogene fusions in invasive mucinous lung adenocarcinoma. *Clin Cancer Res* **2014,** 20, (12), 3087-93.
21. Severson, E.; Achyut, B. R.; Nesline, M.; Pabla, S.; Previs, R. A.; Kannan, G.; Chenn, A.; Zhang, S.; Klein, R.; Conroy, J.; Sausen, M.; Sathyan, P.; Saini, K. S.; Ghosh, A.; Jensen, T. J.; Reddy, P.; Ramkissoon, S. H., RNA sequencing identifies novel NRG1-fusions in solid tumors that lack co-occurring oncogenic drivers. *J Mol Diagn* **2023**.
22. Ptáková, N.; Martínek, P.; Holubec, L.; Janovský, V.; Vančurová, J.; Grossmann, P.; Navarro, P. A.; Rodriguez Moreno, J. F.; Alaghehbandan, R.; Hes, O.; Májek, O.; Pešek, M.; Michal, M.; Ondič, O., Identification of tumors with NRG1 rearrangement, including a novel putative pathogenic UNC5D-NRG1 gene fusion in prostate cancer by data-drilling a de-identified tumor database. *Genes Chromosomes Cancer* **2021,** 60, (7), 474-481.
23. Kohsaka, S.; Hayashi, T.; Nagano, M.; Ueno, T.; Kojima, S.; Kawazu, M.; Shiraishi, Y.; Kishikawa, S.; Suehara, Y.; Takahashi, F.; Takahashi, K.; Suzuki, K.; Takamochi, K.; Mano, H., Identification of Novel CD74-NRG2alpha Fusion From Comprehensive Profiling of Lung Adenocarcinoma in Japanese Never or Light Smokers. *J Thorac Oncol* **2020,** 15, (6), 948961.
24. Liu, Y. F.; Wang, B. Y.; Zhang, W. N.; Huang, J. Y.; Li, B. S.; Zhang, M.; Jiang, L.; Li, J. F.; Wang, M. J.; Dai, Y. J.; Zhang, Z. G.; Wang, Q.; Kong, J.; Chen, B.; Zhu, Y. M.; Weng, X. Q.; Shen, Z. X.; Li, J. M.; Wang, J.; Yan, X. J.; Li, Y.; Liang, Y. M.; Liu, L.; Chen, X. Q.; Zhang, W. G.; Yan, J. S.; Hu, J. D.; Shen, S. H.; Chen, J.; Gu, L. J.; Pei, D.; Li, Y.; Wu, G.; Zhou, X.; Ren, R. B.; Cheng, C.; Yang, J. J.; Wang, K. K.; Wang, S. Y.; Zhang, J.; Mi, J. Q.; Pui, C. H.; Tang, J. Y.; Chen, Z.; Chen, S. J., Genomic Profiling of Adult and Pediatric B-cell Acute Lymphoblastic Leukemia. *EBioMedicine* **2016,** 8, 173-183.
25. Claesson, L.; Larhammar, D.; Rask, L.; Peterson, P. A., cDNA clone for the human invariant gamma chain of class II histocompatibility antigens and its implications for the protein structure. *Proc Natl Acad Sci U S A* **1983,** 80, (24), 7395-9.

26. Strubin, M.; Mach, B.; Long, E. O., The complete sequence of the mRNA for the HLA-DRassociated invariant chain reveals a polypeptide with an unusual transmembrane polarity. *Embo j* **1984,** 3, (4), 869-72.
27. Strubin, M.; Berte, C.; Mach, B., Alternative splicing and alternative initiation of translation explain the four forms of the Ia antigen-associated invariant chain. *EMBO J* **1986,** 5, (13), 3483-8.
28. O'Sullivan, D. M.; Larhammar, D.; Wilson, M. C.; Peterson, P. A.; Quaranta, V., Structure of the human Ia-associated invariant (gamma)-chain gene: identification of 5' sequences shared with major histocompatibility complex class II genes. *Proc Natl Acad Sci U S A* **1986,** 83, (12), 4484-8.
